# A population health approach to workplace mental health: rationale, implementation and engagement

**DOI:** 10.3389/fpubh.2024.1336898

**Published:** 2024-04-18

**Authors:** Kaylee T. Woodard, Allison M. Bailey, Aaron I. Esagoff, Maren S. Fragala, Joanna I. Hayward, Jennifer L. Hunter, Yea-Jen Hsu, Paul M. Kim, Matthew E. Peters, Susan M. Carr

**Affiliations:** ^1^Louisiana State University Health Sciences Center—New Orleans, New Orleans, LA, United States; ^2^Department of Psychiatry and Behavioral Sciences, Johns Hopkins University School of Medicine, Baltimore, MD, United States; ^3^Quest Diagnostics, Secaucus, NJ, United States; ^4^BHS, Baltimore, MD, United States; ^5^emVitals, Cleveland, OH, United States; ^6^The Johns Hopkins University Bloomberg School of Public Health, Baltimore, MD, United States; ^7^Johns Hopkins Healthcare, Baltimore, MD, United States

**Keywords:** employee health, mental wellbeing, mental health risk, proactive outreach, engagement (involvement), assessment

## Abstract

**Objectives:**

To describe a population health-based program to support employee and dependent mental health and learn from engagement trends.

**Methods:**

Retrospective analysis of a program utilizing an assessment of mental health risk. For scoring “at risk,” a Care Concierge is offered to connect users with resources.

**Results:**

Participation was offered to 56,442 employees and dependents. Eight thousand seven hundred thirty-one completed the assessment (15%). Of those, 4,644 (53%) scored moderate or higher. A total of 418 (9%) engaged the Care Concierge. Factors that negatively influenced the decision to engage care included bodily pain, financial concerns. Positive influences were younger age, high stress, anxiety, PTSD and low social support.

**Conclusion:**

Proactive assessment plus access to a Care Concierge facilitates mental healthcare utilization. Several factors influence likelihood to engage in care. A better understanding of these factors may allow for more targeted outreach and improved engagement.

## Application of findings

- Shift workplace culture from reactive to proactive by offering regular mental health risk assessments for all.
- Learn how to create an outreach effort on mental health risk through our story.
- Set expectations for your own population’s mental health engagement rates by risk type based on these results.

## Introduction

The national burden of mental illness is expansive. In 2019, the prevalence of any mental illness in the United States was 20.6%, corresponding to a population of 51.5 million adults, and in 2020, this figure rose to 21%, representing 53 million adults (1). Although caution should be exercised when comparing 2020 prevalence data to years prior, due to the coronavirus disease 2019 (COVID-19) pandemic, an upward trend in mental illness prevalence is noteworthy and plausible (2). On the individual level, one in two adults will meet diagnostic criteria for a mental illness at some point across the lifespan (3). Moreover, common mood disorders often occur comorbidly with substance use disorders and anxiety disorders and other chronic conditions, suggesting that many individuals who struggle with mental illness are hindered by multiple conditions simultaneously. These realities reflect a growing public health crisis that generates significant costs for individuals, businesses, and society at large.

Direct spending on mental and behavioral healthcare in the United States has increased at an average annual rate of 4.6% since 2009 and is projected to surpass $280 billion in 2020 (4, 5). In addition to direct costs, mental illness in working adults generates indirect costs related to productivity loss, absenteeism, and presenteeism (6–13). These costs are estimated at over $1 trillion annually (14).

The economic burden of mental illness is not unique to the United States. Globally, mental illness is a leading cause of disability and contributes to more work loss and work impairment than other chronic medical conditions (15, 16). Beyond the financial consequences, mental illness contributes to personal suffering and diminished quality of life which are far more difficult to quantify.

From a clinical perspective, most mental illnesses are treatable. Multiple lines of evidence also suggest that public investment in mental healthcare has favorable benefit-to-cost ratios and produces cost savings in the long-term (17, 18). Despite the clear benefits of treatment, access to adequate mental healthcare remains profoundly limited for the majority of adults who need it. In multiple national health surveys, millions of adults with mental illness symptoms have reported unmet healthcare needs, leading to socioemotional impairment and reduced functional capacity (4, 19). There is a clear and critical need for concerted public health strategies that promote identification and management of mental and behavioral health conditions. With greater than 60% of the American population participating in the labor force, the workplace represents an opportune environment to trial such initiatives (16, 20).

Many employees have access to an Employee Assistance Program (EAP) as part of their employee benefits. However, utilization of these plans is low (21, 22). Often, they are perceived as crisis support tools rather than a comprehensive mental health initiative for employers. New, more expansive, employer-sponsored mental health programs are emerging. While novel and varied, early data support their use for both employers and employees. Multiple studies suggest that such mental health programs can improve psychological wellbeing and productivity, reduce absenteeism and healthcare costs, and positively shift employer attitudes toward mental health (16, 23–31). While these outcomes are encouraging, the cumulative evidence base for workplace mental health investment remains limited. The programs offered vary widely in approach and offerings. More research is needed on program structure and delivery to inform consensus and best-practice guidelines.

In 2018, Johns Hopkins Healthcare Solutions launched a novel employee mental health engagement program called *Balance.* This program is a proactive, population health-based approach. It delivers a technology-based assessment to all employees and adult dependents that identifies mental health risk factors among users. *Balance* then provides a personal Care Concierge services to bridge users to treatment based on individual needs and preferences using the employee’s own benefits. The Care Concierge team was comprised of trained mental health professionals who were provided access to the *Balance* users benefits in order to guide and advise on care options and resources while also providing in the moment support. The purpose of *Balance* is (1) to improve workplace culture on the topic of mental health by recommending that all employees proactively check-in on their emotional wellbeing on a regular basis and (2) to lower barriers to mental healthcare such as limitations on access, convenience, cost and time requirements to find care. Based on data derived from a preliminary cohort study of *Balance* participants (32), we anticipated strong utilization of the program. In this study, we describe the rationale, development, and implementation of *Balance*, and explore how *Balance* users engaged with the program.

## Method

*Balance* is a novel, employer-initiated employee mental health engagement program. It uses a technology-based behavioral health and wellbeing assessment to build individualized mental health risk profiles for employees. For those who reach a scoring threshold, *Balance* offers “Care Concierge” services to connect employees with appropriate treatment and resources. *Balance* was developed in 2018 at Johns Hopkins as a collaboration between psychiatrists and Johns Hopkins Healthcare Solutions, which is an innovation team within Johns Hopkins Medicine that develops and manages partnerships between healthcare researchers and industrial entities. *Balance* was made commercially available in 2019.

### Eligibility, recruitment, and consent for outreach

This retrospective cohort study reviews data derived from a large national health service company that purchased *Balance* for internal use in 2019. This company employs approximately 46,000 adults across 49 states. Adults with eligible dependents over age 18, such as spouses or adult children, for whom the employees elected to provide medical coverage, were also eligible for *Balance*.

*Balance* was promoted to employees through multiple communications. Employees and eligible dependents who opted to enroll were directed to the *Balance* website, where they received additional information about the program and followed prompts to complete the initial enrollment step. The *Balance* website was accessible wherever the internet was available including at work or at home, and on computers, smartphones, and other portable electronic devices. Enrollment was cost-free, voluntary, and confidential. Registrants were matched to an employer provided eligibility file before receiving access to the *Balance* assessment.

### Study data collection procedures

Data were collected from employees and eligible adult dependents who enrolled in *Balance* during the 6 months between September 4, 2019 and March 6, 2020. The cut-off date of the study period was selected retrospectively to avoid confounding influences related to the effects of the COVID-19 pandemic on mental health symptoms and healthcare utilization. The close date represents the day before significant workplace changes were announced to employees, including work from home policies and temporary furloughs.

Data were collected and deidentified prior to retrospective review. The Johns Hopkins University Institutional Review Board determined that this research qualified as exempt under Department of Health and Human Services regulations based on its use of deidentified data for secondary research without intention to reidentify or contact study participants (IRB00220549).

### Data monitoring and quality control

Demographic and clinical data were collected and stored electronically on a secure web-based platform in accordance with the Health Insurance Portability and Accountability Act (HIPAA). Data quality was optimized by the use of standardized electronic data collection forms. Participant responses were restricted to automated input fields and fixed options to ensure consistency across participants and worksites, and to avoid clerical errors.

Data regarding referral patterns, health care utilization, and time spent coordinating care on the behalf of *Balance* users were collected by the Care Concierge. The eligibility data file, the assessment data, and the Care Concierge data were then concatenated into one file for analysis.

### Behavioral health and wellbeing assessment

After enrolling in *Balance,* employees completed a standardized electronic behavioral health and wellbeing assessment offered through a virtual platform. The assessment consisted of 48 questions evaluating mental health symptom burden. Question topics included medical history, depression, anxiety, substance use, sleep, and stress. Employees were able to complete the assessment in one sitting or save their responses for completion at a later point in time.

Assessment questions on medical symptom burden focused on the presence or absence of chronic medical conditions including chronic pain. Assessment questions on mental health symptom burden were compiled from validated, self-administered psychiatric symptom scales, including the Patient Health Questionnaire-9 (PHQ-9), the Generalized Anxiety Disorder-7 (GAD-7), the Perceived Stress Scale-4 (PSS-4), the abbreviated PTSD Checklist-Civilian Version (PCL C-6), the Alcohol Use Disorders Identification Test (AUDIT-C), and The Oslo Social Support Scale.

### Feedback report and risk stratification

The assessment scores on all measures were calculated and an overall acuity score was generated. This acuity score served as a gate-keeper to the Care Concierge with those scoring at moderate or above overall risk encouraged to schedule a consult with the Care Concierge immediately upon completing the assessment.

Individualized summary reports for each participant based on his/her assessment responses were provided immediately to the *Balance* user. These reports included feedback on eight specific domains of mental health and wellbeing, as well as an overall mental health risk group assignment. Domains included depression, anxiety, stress, alcohol misuse, substance misuse, sleep, social determinants of health, and life events. A more detailed report was also sent to the Care Concierge for those *Balance* users scoring moderate or above on overall mental health risk.

- *Mental health*: symptom burden was classified categorically from “no risk” to “severe risk” for four domains of mental health including depression, anxiety, traumatic stress, and alcohol use. For each of these domains, participants were given a visual representation of their symptom burden in the form of a color-coded graphic, along with a brief summary comment and/or a recommendation for next steps.
- *Wellbeing*: the wellbeing profile was classified categorically from “low” to “high” for four common measure of overall wellbeing including, perceived stress, financial stress, social support, and sleep disturbance. For each of these factors, participants were given a visual representation of their symptom burden in the form of a color-coded graphic, along with a motivational comment and/or a brief recommendation for stress-reduction.
- *Overall risk group stratification*: in order to focus utilization of Care Concierge services on those with the greatest need, an overall risk group categorization was used. The goal of the overall risk assignment was to capture all *Balance* users who may benefit from a Care Concierge consult with priority going to those with the greatest need. The algorithm to calculate overall risk from the various assessments of specific domains was tested to ensure that any *Balance* user who scored moderate or above for any one of several key inventories such as depression, anxiety, substance use, alcohol misuse, and traumatic stress were captured as moderate or higher on the overall mental health risk score. The overall risk algorithm reflected the risk levels of the validated, standardized measures included in the assessment.

### Care Concierge outreach process

After submitting the assessment but before receiving feedback, employees were asked if they would like to receive outreach from a mental healthcare professional if their results indicate a need. After receiving results, a link was provided to self-schedule with the Care Concierge for all those scoring at a moderate or above risk level for overall acuity. For those who did not self-schedule and who elected to receive outreach, the Care Concierge could then reach out via e-mail or phone to schedule an appointment to discuss results and explore options for care. Those who did not consent to outreach but scored moderate or higher in risk were provided the link and encouraged to self-schedule a Care Concierge appointment. This approach allowed all *Balance* users to opt out of outreach for those disinclined to receive it. It also afforded those comfortable with outreach with multiple touch points to schedule an appointment.

### Referrals, resources, and bridging to care

The Care Concierge received full assessment results from all *Balance* users who scored moderate or higher. Appointments were conducted by phone. The initial appointment was approximately 40 min in length and included a review of the assessment results as well as a few additional questions. Answers were recorded in an Electronic Medical Record (EMR) and were later concatenated to the assessment data and eligibility data file for a complete record of the *Balance* user’s engagement with the program. The Care Concierge team was comprised of masters degree-level mental health practitioners such as social workers and therapists, trained on all relevant benefits information for the employer, then discussed resources and care options with *Balance* users to determine how best to engage in mental health care. At the completion of each appointment, a care plan was developed and issued to the *Balance* user to guide them to resources. Resources suggested in the care plans included referral to a behavioral health provider through EAP for short term issues, the health insurance provider for long term needs, local emergency resources, and support groups. In addition, tip sheets were offered on ways to connect to a medical provider (e.g., a primary care provider). Also offered were specific financial resources available through the employer and ways to take advantage of work life services (e.g., childcare, eldercare, or financial resources). When behavioral health treatment from an EAP or medical provider was recommended and agreed to by the user, the Care Concierge facilitated access to available providers who were accepting taking new patients and who met the user’s criteria (location, personal preferences, insurance, and required skill sets). The time necessary to identify resources was tracked by the Care Concierge for later analysis.

Outreach efforts by the Care Concierge to secure appointments with those scoring moderate or higher were also tracked by channel of outreach (i.e., e-mail, phone call) and frequency.

### Data capture

Participation and utilization data included:

- *Concierge outreach efforts*: as measured by the count of phone calls and/or e-mails to secure initial connection to the Care Concierge;
- *Employee participation patterns*: measured as the percentage of employees and eligible dependents who completed enrollment in *Balance* and submitted the behavioral health and wellbeing assessment;
- *Care Concierge utilization*: measured as the percentage of employees who were eligible for a Care Concierge consultation based on risk stratification, and subsequently completed a Care Concierge consultation;
- *Care Concierge engagement*: measured by time spent working with *Balance* users, categorized by time spent in consultation with the user and time spent engaged in outreach to secure appointments with referred resources; and
- *Completed referrals* recorded in the Care Concierge record when *Balance* users responded to queries from the Care Concierge confirming successful completion of the first appointment with a provider.

Data used to predict use of concierge services included:

- *Baseline demographics*: age and gender;
- *Medical history*: presence or absence of chronic medical conditions including chronic pain;
- *Self-reported mental and behavioral health symptoms*: as measured by the PHQ-9, GAD-7, PSS-4;
- *Social determinants of health*: as measured by the Oslo Social Support Scale, and a series of questions that align with the Center for Disease Control and Prevention’s (CDC) social determents of health including: food and shelter insecurity, risk for physical and verbal abuse, caring for a loved one with a mental or physical illness and job loss or insecurity; and
- *An overall risk-stratification score*: as calculated by a proprietary clinical scoring algorithm that incorporated measurements of mental and behavioral health history and social determinants of health as defined above for the purpose of determining need and access of Care Concierge services

### Analysis

We describe the number of employees and dependents who completed the behavioral health and wellbeing assessment, what their risk scores were, and whether they used the Care Concierge services. We then describe the characteristics of individuals who scored moderate risk or higher and who used or did not use the concierge services, reporting frequencies with percentages for categorical variables and mean with interquartile ranges for continuous variables. We conducted bi-variate analysis to examine the association between each predictive variable with use of concierge services using chi-square tests or Wilcoxon rank sum tests. To further explore the driving factors for individuals to use the concierge services, we performed logistic regression models using the backward stepwise selection of variables with elimination criteria set at the *p* = 0.2 level. The stepwise approach is a method used to fit regression models by iteratively selecting predictive variables based on predefined criteria. In each step, variables are considered for inclusion or exclusion from the model, with a prespecified criterion, typically set at a significance level of *p* = 0.2 as utilized in this study. This iterative process is particularly useful for exploratory analyses, allowing for the management of a large number of potential predictor variables and the selection of the most appropriate set for model inclusion. The predictive variables included in the model have been described in the data capture section. Moreover, the criterion level of *p* = 0.2 was chosen to balance the need to limit information loss while dealing with a substantial number of predictive variables (>25 in this study) (33–35). This threshold aligns with commonly adopted practices in stepwise regression modeling. It’s important to note that the significance level was set at *p* = 0.05.

For employees and dependents who used the concierge services, we described whether an action plan was created, whether the case was closed, overall duration of activities, number of appointments, outreach activities, and referrals recommended and used. Several *Balance* users took the *Balance* assessment multiple times throughout the time period. Our analysis focused on initial assessments only. Two employees presented to the Care Concierge based on references without first taking the *Balance* assessment.

## Results

Of the 56,442 employees and dependents who were offered to participate in *Balance* during the time period 11,567 registered for *Balance* (21%), and 8,731 completed the assessment (15% of all those eligible). A total of 4,644 employees (53% of those who completed the assessment) scored moderate risk or higher and were proactively offered Care Concierge services after completing the survey.

Of those who completed the assessment, 70% indicated their willingness to be contacted by the Care Concierge if their results indicated they may benefit from a consultation. Among the 418 with moderate or high risk and used concierge services, 215 (51%) self-scheduled their appointment “prior to outreach” while 192 (46%) scheduled in response to outreach.

Among those who had access to the Care Concierge (4,644), several differences in likelihood to follow-up with the service were noted (Table 1). Age group trends were evident. Younger *Balance* users were more likely to schedule and complete a follow-up consultation (ages 19–34 years) compared to other age groups (*p* = 0.002). However, those age 45–64 scored at a similar rate to the youngest age group (28.5% engaging in follow-up compared to 29% in the youngest group). *Balance* users currently seeing a doctor, therapist, or other professional for a mental health concern were more likely to complete a consultation with the Care Concierge (*p* = 0.010) and those who consented to outreach from the Care Concierge were more likely to engage and complete a Care Concierge appointment (*p* < 0.0001).

**Table 1:** *Balance* users scoring moderate or above in mental health risk who elected to engage (yes) or not engage (no) the Care Concierge follow-up service.

	Follow-up consultation				*p*-value
	No		Yes		
	*n* = 4,212	%	*n* = 418	%	
*Age*					
19–34	1,267	30.1	121	29.0	0.002
35–44	1,114	26.5	82	19.6	
45–54	1,062	25.2	119	28.5	
55–64	657	15.6	88	21.1	
65+	112	2.7	8	1.9	
*Gender*					
Male	936	22.2	80	19.1	0.338
Female	3,253	77.2	336	80.4	
Non-binary or transgender	23	0.6	2	0.5	
*Active military member or veteran*					
No	4,073	96.7	406	97.1	0.637
Yes	139	3.3	12	2.9	
*Screen of drug use*					
Negative	3,954	93.9	392	93.8	0.939
Positive	258	6.1	26	6.2	
*Currently seeing a doctor, therapist or other professional for a problem*					
No	3,200	76.0	298	71.3	0.034
Yes	1,012	24.0	120	28.7	
*Asthma*					
No	3,545	84.2	353	84.5	0.879
Yes	667	15.8	65	15.6	
*Back pain*					
No	1991	47.3	190	45.5	0.478
Yes	2,221	52.7	228	54.6	
*Cancer*					
No	4,057	96.3	402	96.2	0.879
Yes	155	3.7	16	3.8	
*COPD*					
No	4,128	98.0	406	97.1	0.230
Yes	84	2.0	12	2.9	
*Diabetes*					
No	3,852	91.5	379	90.7	0.586
Yes	360	8.6	39	9.3	
*Heart disease*					
No	4,126	98.0	409	97.9	0.878
Yes	86	2.0	9	2.2	

Chi square tests were used to identify demographics and healthcare related factors that were a statistically significant impact in whether or not a follow-up visit was completed. *Balance* users currently seeing a provider for a mental health issue were more likely to complete a follow-up visit. Those between age 19–34 were more likely to engage the Care Concierge as well. Fourteen *Balance* users were excluded due to missing age or gender information.

Eligibility to meet with the Care Concierge was defined by an overall mental health risk score designed to ensure that Care Concierge resources were granted to those most in need. The choice to engage the Care Concierge was associated with certain trends in the screening tool. Individuals who engaged the Care Concierge scored higher on the PSS, GAD2, GAD7, PHQ8, PCL-C-2 AND PCL-C-5 and lower on the Oslo Social Support Scale, generally signifying higher perceived stress, more severe anxiety and depression, greater severity of trauma symptoms, and fewer social connections and support systems (Table 1).

We examined indicators useful in measuring social determinants of health among *Balance* users based on Oslo Social Support Scale and a series of questions that align with the CDC’s social determents of health to understand likelihood to engage in services based on lifestyle factors. Responses that were indicators of increased likelihood to access the Care Concierge are listed in Table 2 and these included experiencing verbal abuse (*p* = 0.002), experiencing a financial crisis in the past 6 months (*p* = 0.031), and having financial concerns regarding meeting daily needs (*p* = 0.031) and monthly expenses (*p* = 0.014).

**Table 2:** Social determinants of health (SDoH) factors between users with vs. without follow-up.

	Follow-up consultation				*p*-value
	No (*n* = 4,212)		Yes (*n* = 418)		
How often does anyone, including family, insult or talk down to you? Mean (SD)	2.24	1.09	2.43	1.16	0.001
How often does anyone, including family, physically hurt you? Mean (SD)	1.08	0.35	1.06	0.33	0.069
How often do you worry about money? Mean (SD)	1.82	1.11	1.87	1.10	0.420
*I have had a financial crisis in the last 6 months, n (%)*					
Agree	1813	43.0	158	37.8	0.039
Disagree	2,399	57.0	260	62.2	
*My greatest financial concern, n (%)*					
Meeting daily needs such as food and shelter	1,129	26.8	78	18.7	<0.001
Meeting my monthly expenses such as car payments and insurance	2,276	54.0	201	48.1	0.020
Paying off debt	2,840	67.4	281	67.2	0.933
Saving for retirement	2,436	57.8	256	61.2	0.178

Social determinants of health of *Balance* users were measured by indicators listed in this table. Wilcoxon rank sum tests or chi square tests were used to determine which variables were significant in affecting the likelihood of engagement in follow-up visits. Individuals completing a follow-up visit were more likely to be experiencing verbal abuse and less likely to be in a financial crisis.

A stepwise regression analysis addressed potential confounding variables such as age and consent for Care Concierge contact (Table 3). Factors that negatively influenced the decision to engage the Care Concierge included: bodily pain in the last month, meeting monthly expenses (payments and car insurance), positive drug screen, financial crisis in the last 6 months, tobacco use, and meeting daily needs such as food and shelter. Factors that positively influenced the decision to engage the Care Concierge included: being between the ages of 45–64, higher levels of stress, and reporting symptoms of anxiety and PTSD.

**Table 3:** Stepwise regression on factors that contributed to the likelihood to engage in Care Concierge follow up after assessment results.

	Outcome variable: engagement in Care Concierge follow up (*n* = 4,630)		
	Odds ratio	95% CI	*p*-value
Age			
19–34	1		
35–44	0.91	(0.67–1.22)	0.517
45–54	1.47	(1.11–1.94)	0.006
55–64	1.74	(1.28–2.36)	<0.001
65+	0.92	(0.43–1.96)	0.828
Physically hurt	0.78	(0.56–1.10)	0.155
Financial crisis in the last 6 months	0.76	(0.59–0.97)	0.030
Have concerns on meeting daily needs such as food and shelter	0.69	(0.52–0.93)	0.014
Have concerns on meeting my monthly expenses such as car payments and insurance	0.83	(0.65–1.05)	0.125
Perceived stress score (PSS)	1.11	(1.06–1.16)	<0.001
Generalized anxiety disorder (GAD)-2	1.09	(1.01–1.17)	0.022
PCL-C-2 score	1.07	(1.01–1.12)	0.011
Oslo social support scale (OSSS-3)	0.95	(0.91–0.99)	0.026
Tobacco use	0.94	(0.87–1.02)	0.147
Bodily pain in the past month	0.87	(0.80–0.95)	0.002

A stepwise regression on follow-up consultations was done to address confounding variables (consent for Care Concierge contact and visits done after March 6th 2020). This table lists, the demographics, social determinants of health and screening surveys measured in the analysis.

The mean number of outreach activities was 2.43 attempts per *Balance* user (Table 3). On average, a Care Concierge spent 2.5 h per person in a follow-up visit with a standard deviation of 1.28 h. If employees presented to the Care Concierge with high emotional affect or specific requests in care management, visits lasted longer (Table 4).

**Table 4:** Description of activity hour, appointments, and outreach activities (*n* = 418).

	Mean	SD	Median	IQR	Range	% individuals with at least one appointment or outreach activities
Activity hour	2.53	1.28	2.25	(1.58–3.08)	(0.58–9.58)	
*Appointment*						
No. of all appointments	1.09	0.30	1	(1–1)	(1–3)	100
No. of telephonic appointments	1.02	0.39	1	(1–1)	(0–3)	94.0
No. of video appointments	0.06	0.26	0	(0–0)	(0–2)	6.2
*Outreach activity*						
No. of outreach activities	2.43	1.61	2	(1–3)	(0–15)	94.3
No. of emails	1.30	0.68	1	(1–2)	(0–4)	89.0
No. of guide activities	0.01	0.11	0	(0–0)	(0–2)	0.5
No. of independent activities	0.00	0.05	0	(0–0)	(0–1)	0.2
No. of legacy activities	0.36	0.72	0	(0–1)	(0–6)	26.1
No. of phone calls	0.77	0.81	1	(0–1)	(0–5)	59.1

Care Concierges tracked time spent with *Balance* users during follow-up visits as well as the methods and time spent on outreach efforts for those who consented to outreach. The average time spent with employees was 2.53 h with a notably wide range of 0.58–9.58 h, highlighting the ability of balance to deliver individualized care. Approximately 2.4 outreach activities were employed by Care Concierges to engage *Balance* users who consenting to be contacted to complete follow-up visits.

The Care Concierge created care plans for 393 participants. Those care plans included 281 referrals to other mental health resources and providers. The number of referrals made in the care plans ranged from 0 to 4. A total of 252 participants followed up for multiple conversations with their Care Concierge for in-the-moment support or follow-up on the care plan and referrals. Care Concierges provided at least one recommendation to 94% of *Balance* users they engaged (Table 5). Emotional wellbeing, establishing a short-term provider, and referrals to behavioral health/substance misuse providers were the most common recommendations at 78, 55.3 and 24.6%, respectively. Referrals pertaining to substance abuse facilities, behavioral health facilities, and career wellbeing were among the least utilized during Care Concierge visits at 0, 0.2 and 0.2%, respectively.

**Table 5:** Description of recommendations/referrals and usage rate (*n* = 418).

	Mean	SD	Median	IQR	Range	% individuals with at least one recommendation/referral	% individuals with referrals using at least one recommendation/referral
No. of all recommendations/referrals	2.03	1.13	2	(1–3)	(0–6)	94.0	60.3
Behavioral health facility	0.00	0.05	0	(0–0)	(0–1)	0.2	0.0
Behavioral health/substance abuse provider	0.31	0.59	0	(0–0)	(0–3)	24.6	3.9
Career wellbeing	0.00	0.05	0	(0–0)	(0–1)	0.2	0.0
Emotional wellbeing	0.81	0.47	1	(1–1)	(0–2)	78.0	67.8
Financial wellbeing	0.16	0.46	0	(0–0)	(0–2)	11.5	33.3
Physical wellbeing	0.05	0.26	0	(0–0)	(0–3)	3.8	0.0
Short term provider	0.61	0.62	1	(0–1)	(0–3)	55.3	11.7
Substance abuse facility	0.00	0.00	0	(0–0)	(0–0)	0.0	—
Support group	0.04	0.20	0	(0–0)	(0–2)	3.6	13.3
Work life services	0.03	0.19	0	(0–0)	(0–2)	2.4	20.0

Utilization of recommendations by the Care Concierge team by *Balance* users are noted in this table. Ninety-four percent of employees completing a follow-up visit received at least one recommendation from the Care Concierge. The most frequently used recommendations provided to users include those pertaining to addressing emotional wellbeing, establishing a short term provider and seeking behavioral health/substance abuse providers. Sixty percent of users receiving a recommendation were recorded as using at least one of the recommendations provided by the Care Concierge. The most utilized recommendation pertained to addressing emotional wellbeing, financial wellbeing and work life services.

Of the recommendations given, the degree to which a type of referral was utilized by a *Balance* participant was measured as the percentage of individuals using the recommendation provided (Table 5). Of the employees who received at least one recommendation, 60.3% were recorded as using at least one of the referrals provided by the Care Concierge. Emotional well-being, financial wellbeing, and work life services were the most utilized recommendations by users at 67.8, 33.3, and 20%, respectively. Of the 418 Care Concierge engagements, 298 individuals (71%) reported that they were seeking mental health services for the issue at hand for the first time while 120 (29%) were seeking support for an issue where a mental health provider was currently engaged.

## Discussion

The Johns Hopkins *Balance* program is designed to support employers and employees by raising awareness of mental health risk and facilitating care for those in need. In doing so, the program collects valuable data on the mental health challenges of participants as well as the way in which these individuals choose to engage with mental healthcare resources. By analyzing data collected from employees and eligible dependents enrolled in *Balance*, this retrospective cohort study investigates the utilization of a novel mental health engagement program. Results demonstrate differences in willingness to engage mental health services by a variety of factors. By understanding who may be inclined or disinclined to engage in care we may be able to tailor strategies to the special needs of each group.

In total, 8,731 employees and eligible dependents completed the *Balance* screening assessment. Of those, 418 *Balance* users scoring moderate or above in overall acuity completed a visit with the Care Concierge service. The Care Concierge is a trained and licensed mental health professional receiving guidance and oversight from Johns Hopkins *Balance* Medical Directors. For the 418 *Balance* participants who engaged the Care Concierge, a phone consultation took place to review *Balance* assessment results and to discuss current mental health needs and available resources.

*Balance* was designed with the understanding that (1) mental healthcare is valuable and effective and (2) the process of securing mental healthcare is time-consuming and difficult. By proactively encouraging a population to assess their mental health status and by making the connection to care markedly easier for those in need, employers can meaningfully impact the health of employees and their dependents.

*Balance* was designed to improve workplace culture related to mental health and to lower the barriers to care for those in need. Proactive efforts may prevent expensive acute episodes of care such as emergency room visits or inpatient hospitalizations, as well as improving employee performance via reduced absenteeism and increased presenteeism (36). *Balance* is distinguished from other employee health resources currently offered in the workforce by using a proactive, population health-based approach for all employees at every level of the organization and at every level of mental health risk.

When studying utilization data, several variables were found to be associated with likelihood to engage the Care Concierge for those scoring at moderate or above overall risk. A bimodal distribution was evident by age. Participants whose age fell into the 19–34 range were significantly more likely to complete a meeting with the Care Concierge (29%). This may be an indication of increased openness, willingness, need or availability to engage in mental health. Similarly, rates of Care Concierge use for 45–64 year old age group was high (28.5%) This age range may represent individuals who increased availability to seek out mental healthcare due to changes in personal responsibilities. This age range may also include the accumulation of pressing life events which may explain the increased participation in Care Concierge consultations. Users between 35–44 were less likely to engage in the Care Concierge. There are several possible explanations including increased use of other care options outside of the *Balance* Care Concierge, less time to engage in mental healthcare due to other life responsibilities, or less appetite to engage in mental healthcare services. The multimodal age distribution illustrates *Balance*’s potential to provide resources intrinsic to employees as they move through different stages of life.

In addition to demographic patterns, we saw patterns in the relationship between type of mental health risk and the choice to engage the Care Concierge. Those experiencing heightened levels of perceived stress and increased severity of anxiety and PTSD symptoms were more likely to engage the Care Concierge than others. This may suggest that these conditions motivate the individual to seek care compared to other conditions. The cause for this observation is unknown, but a possible explanation may be that employees with these anxious symptoms may more likely seek help because they believe that these symptoms can be effectively treated with medications and therapies compared to other psychiatric symptoms. Individuals who reported lower social support scores were more likely to engage the Care Concierge. This may suggest that those with stronger social networks are less likely to engage in mental health resources than those who are more isolated.

Variables decreasing the likelihood of employees following up with a Care Concierge include substance use disorder, bodily pain, and presence of financial crisis. Substance use disorders may be substantively different from other mental health conditions in a variety of ways. Stigma may be a major barrier, particularly in the workplace. Moreover, treatment for substance use disorders may present the individual with a different risk/reward consideration compared to other mental health treatments. Treatment success may be perceived as less likely or too costly (37, 38). Given the importance of treating addiction, particularly for employers, an approach to better target and support these *Balance* users should be identified.

Similarly, if those experiencing physical pain are less likely to address their mental health issues, we may be losing the opportunity to make an important impact on providing care for employees’ physical and mental needs. Physical pain may cause depression and depression may make one less likely to effectively manage physical pain. Individuals suffering physical pain may attribute their emotional state to their taxing physical pain causing them to overlook the opportunity to address mental health challenges. Identifying those with heterogeneous conditions may allow for targeted outreach and greater engagement in mental health solutions.

A similar phenomenon may be true for those who have undergone a recent financial crisis, as individuals may be less likely to seek mental healthcare because they have prioritized addressing the immediate financial needs rather than investing time and resources into mental health. Offering special programs to support financial stress paired with targeted messaging may help engage these individuals to utilize services addressing the interplay between lifestyle challenges, mental health, and their possible confounding effects.

*Balance* successfully identified individuals with no past or present healthcare providers and offered guidance in connecting them with appropriate resources. Capturing this subset of individuals highlights the program’s accessibility to people who potentially would not receive professional healthcare otherwise. Tailored recommendations addressing the specific needs of each individual user illustrates the breadth and flexibility of *Balance*’s support for employee wellbeing.

While our data did not include *Balance* utilization during the pandemic, preliminary evidence suggests that quarantine and isolation for infection control will be associated with an increased incidence of mental health conditions (2), as mental health sequelae of the coronavirus pandemic will likely only increase the need for employers to encourage and facilitate mental health resources (39). Moreover, the use of technology-based screening tools and telehealth interventions are becoming increasingly relevant as institutions across the country restructure their environments to promote social distancing measures (40).

## Limitations

The *Balance* assessment was completed by 8,731 employees and eligible dependents. Of those, 420 continued on to the Care Concierge service. However, many others may have engaged in mental healthcare on their own without use of the Care Concierge. A future analysis of claims and pharmacy data to track utilization of healthcare services amongst *Balance* users vs. non-users is planned to determine if the assessment alone was useful in driving mental healthcare services.

Once referrals were delivered to the 418 individuals who met with the Care Concierge, confirmation that appointments were kept was not reliably recorded as providing that information was at the discretion of the *Balance* user or service provider. This analysis cannot be used to demonstrate that mental health care services were delivered for all those who accessed the Care Concierge and were referred to care. The outcomes of mental health care services delivered by providers were not captured. Taking the assessment may have encouraged some to seek mental health services from existing providers rather than engaging the Care Concierge offering. Our study did not capture all possible impacts of the assessment process.

*Balance* was designed to improve workplace culture related to mental health and to lower the barriers to care for those in need. However, our study did not allow for a comparison of pre-post workplace culture nor for use of comparison groups. We were able to quantify utilization of the program but could not compare it to mental healthcare in the absence of *Balance*.

## Future offerings

The *Balance* team meets regularly to improve the program based on user feedback and advancements in the field of mental health. The next iteration of *Balance* will include a psychoeducational component that teaches users about mental health etiology and treatment through the lens of the Johns Hopkins perspectives of psychiatry model (41). The *Balance* program is a high-touch model capturing a wide range of personal struggles that influence mental health. Our study demonstrates how a human-centric, scalable program connects its users to resources specific to the needs of each individual. The human touch through the Care Concierge service is central to *Balance*. However, offering additional asynchronous channels of connection and Artificial Intelligence (AI) based tools may allow for improved scale of mental healthcare. These novel approaches recognize that the market for mental healthcare may include preferences for non-traditional tools that are safe and effective to reduce stress and treat common conditions. Further, the first year deploying *Balance* identified the need to support mental health needs of dependent children of employees. The *Balance* assessment was intended for the *Balance* user’s use only; however, the employees often needed Care Concierge support to address the mental health needs of a loved one. Future versions of *Balance* allow Care Concierge access for all dependent children and a channel to the Care Concierge for direct work with adolescents in need.

## Conclusion

Despite improved awareness of mental healthcare needs during and after the COVID-19 pandemic, barriers to mental healthcare continue to persist in many settings. Even in employer populations with generous employee benefits, robust care networks, and a recent trend to offer added care navigation, challenges remain for many individuals in need of mental healthcare. *Balance*’s approach of encouraging mental health risk assessment, coupled with the Care Concierge service, across a large employer population is novel. Additionally, proactively assessing mental health status may prevent future development of a more severe psychiatric condition, as well as improving awareness of mental health risks so that individuals may monitor for any warning signs and symptoms of mental illnesses. Therefore, improved benefit design that combines assessment with care navigation may offer advantages for employers, employees, and their families. Insights into the factors associated with likelihood to engage in care may aid in more targeted and effective approaches to engagement.

## Data availability statement

The datasets presented in this article are not readily available because they are the property of the employer. The data set will not be shared. Requests to access the datasets should be directed to SMCarrMPH@gmail.com.

## Ethics statement

The studies involving humans were approved by IRB: Johns Hopkins University School of Medicine Institutional Review Board exempted (IRB00220549). The studies were conducted in accordance with the local legislation and institutional requirements. Written informed consent for participation was not required from the participants or the participants’ legal guardians/next of kin in accordance with the national legislation and institutional requirements.

## Author contributions

KW: Writing – review & editing, Project administration. AB: Writing – original draft. AE: Writing – review & editing. MF: Writing – review & editing. JoH: Writing – review & editing. JeH: Writing – review & editing. Y-JH: Formal analysis, Data curation, Writing – review & editing, Methodology. PK: Writing – review & editing. MP: Writing – review & editing, Methodology, Conceptualization. SC: Writing – review & editing, Resources, Project administration, Methodology, Conceptualization.
